# Interleukin-17D promotes lung cancer progression by inducing tumor-associated macrophage infiltration via the p38 MAPK signaling pathway

**DOI:** 10.18632/aging.204208

**Published:** 2022-08-05

**Authors:** Zhenzhen Lin, Qiumin Huang, Junrong Liu, Hao Wang, Xuexi Zhang, Zhiyan Zhu, Wei Zhang, Yiliang Wei, Zhe Liu, Wei Du

**Affiliations:** 1 Department of Immunology, Biochemistry and Molecular Biology, 2011 Collaborative Innovation Center of Tianjin for Medical Epigenetics, Tianjin Key Laboratory of Medical Epigenetics, Tianjin Medical University, Tianjin 300070, China; 2 Jiangsu Key Laboratory of Phylogenomics and Comparative Genomics, School of Life Sciences, Jiangsu Normal University, Xuzhou, Jiangsu 221116, China; 3 Key Laboratory of Immune Microenvironment and Disease of the Ministry of Education, Tianjin Medical University, Tianjin 300070, China; 4 Tianjin Key Laboratory of Radiation Medicine and Molecular Nuclear Medicine, Institute of Radiation Medicine, Tianjin 300192, China

**Keywords:** cytokine, IL-17D, lung cancer, MAPK signaling pathway, targeted therapy

## Abstract

Cancer immunoediting is defined as the integration of the immune system’s dual host-protective and tumor-promoting roles, including three phases: elimination, equilibrium, and escape. Immune selective pressure causes tumor cells to lose major histocompatibility complex expression or acquire immunosuppressive gene expression, which promotes tumor immune evasion and tumor progression. Interleukin-17D (IL-17D), a member of the IL-17 family of cytokines, plays an important role in the host defense against infection and inflammation. However, the role of IL-17D in the progression of lung cancer remains unclear. In this study, we found that IL-17D was highly expressed in human lung cancer, and increased IL-17D expression was associated with tumor stage and short overall survival. IL-17D overexpression significantly promoted tumor growth in subcutaneous xenograft mouse models but only slightly affected cell proliferation *in vitro*. Using flow cytometry, we found that IL-17D overexpression enhances the recruitment of tumor-associated macrophages to the tumor microenvironment. Based on the expression profile of *IL17D*–overexpressing A549 cells, we found that IL-17D increased the expression levels of macrophage polarization– and recruitment–related genes through the MAPK signaling pathway. Moreover, inhibition of the p38 pathway blocked macrophage infiltration induced by IL-17D. These results suggest that IL-17D regulates the tumor immune microenvironment via the p38 MAPK signaling pathway, highlighting IL-17D as a potential therapeutic target for lung cancer.

## INTRODUCTION

The immune system plays a dual role in cancer development and progression, including both host protection and tumor promotion. Cancer cells express antigens that differentiate them from non-transformed cells. Immune responses to tumor antigens initiate three sequential phases: tumor cell elimination, equilibrium, and escape under immunosurveillance, called “cancer immunoediting” [1, 2]. Under immune selective pressure, tumor cells gain resistance against the immune system, via mechanisms involving the expression of inhibitory ligands or immunosuppressive cytokines and loss of antigens, leading to immune evasion and immunotherapy failure [3].

The functions of the IL-17 family in regulating immune response have been linked to many disease processes, including rheumatoid arthritis and antitumor immunity [4–8]. IL-17D, as a novel member of the IL-17 family, could regulate local immune responses through inducing cytokine production [8]. Unlike other family members, IL-17D is unusually expressed outside the immune system. Based on the expression pattern (RNA-seq) [9] and quantitative real-time polymerase chain reaction (qRT-PCR) [10], IL-17D is preferentially expressed in the brain, ovary, skeletal muscle, adipose tissue, heart, lung, and pancreas. Previous studies have revealed that IL-17D can stimulate endothelial cells to produce IL-6, IL-8, and granulocyte-macrophage colony-stimulating factor [10]. However, the role of IL-17D in antitumor therapy remains controversial. O’Sullivan et al. used fibrosarcoma model and found that IL-17D inhibited tumor progression via NK-cells [11]. Recent report indicated that *Il17d^−/−^* mice were resistant to tumor growth upon B16 OVA melanoma and E.G7 OVA lymphoma challenge through enhancing CD8 T cell activity [12]. These suggest that IL-17D may exhibits a variety of functions in different tumor types and stages of tumor development. Here, we study the molecular and clinical consequences of IL-17D expression in lung cancer.

In this study, we sought to identify tumor-expressed genes that play an important role in tumor progression. We found that IL-17D was highly expressed in lung cancer and associated with poor clinical outcomes. Mechanically, IL-17D induced tumor associated macrophages (TAMs) infiltration via the p38 MAPK pathway. These data delineate an important role for IL-17D in the immune microenvironment of lung cancer, and suggest that IL-17D might be a potential target for the treatment of lung cancer.

## RESULTS

### IL-17D expression correlates with poor prognosis in human lung cancer

**Figure 1:**
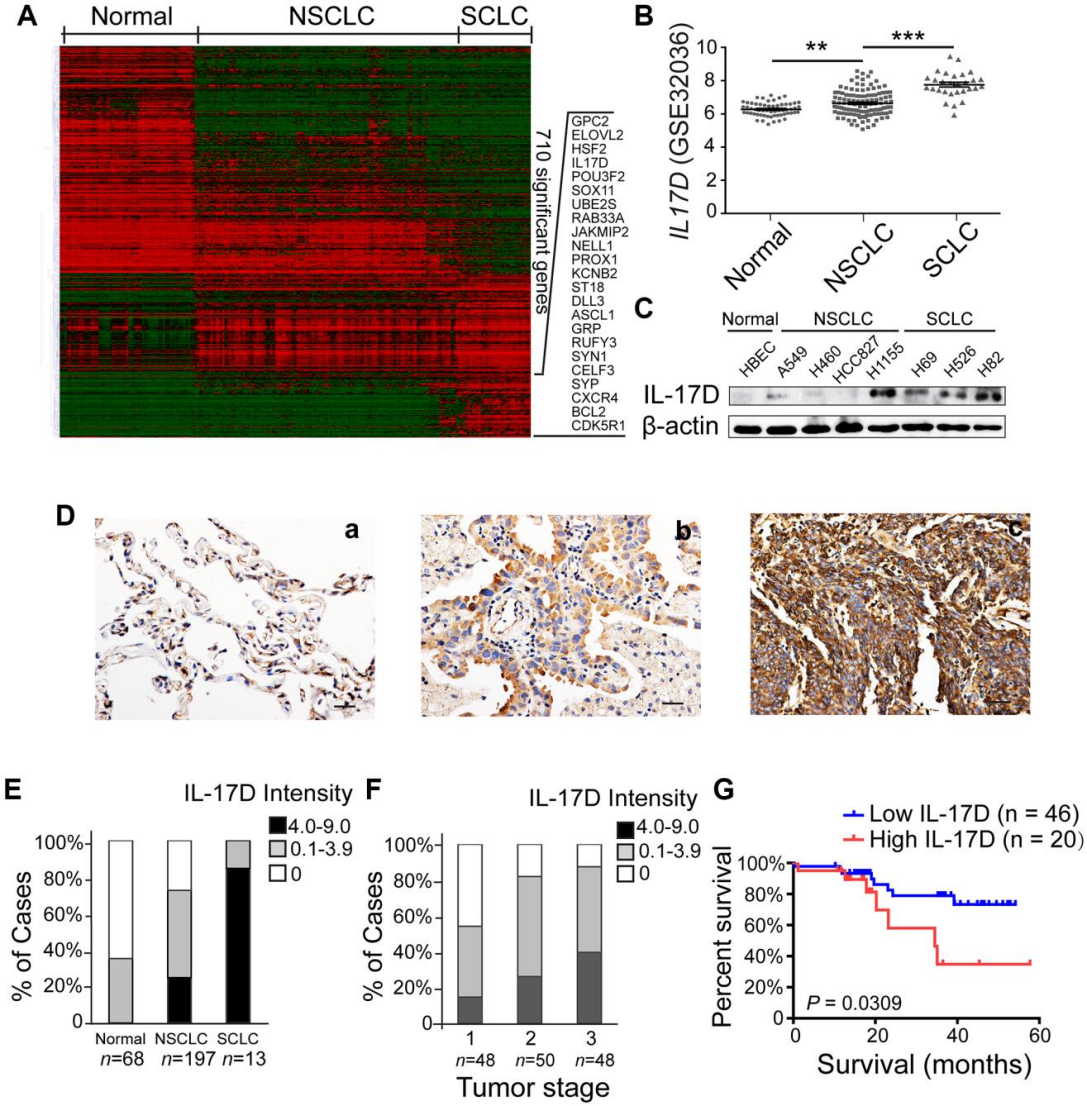
**Interleukin (IL)-17D is highly expressed in lung cancer cell lines and tissues and correlates with poor prognosis in human lung cancer.** (**A**) Heatmap of 856 differentially expressed transcripts (710 genes) among normal airway epithelial cell lines (Normal, *n* = 59), non-small cell lung cancer cell lines (NSCLC, *n* = 118), and small cell lung cancer cell lines (SCLC, *n* = 29). (**B**) RNA-seq data (GSE32036) of *IL-17D* gene expression of normal airway epithelial cells (*n* = 59) and NSCLC (*n* = 118) and SCLC (*n* = 29) cell lines (value = quantile-normalized and log_2_-transformed signal). ^**^P < 0.01. ^***^P < 0.001. (**C**) Immunoblotting was performed to examine the expression level of IL-17D in normal, NSCLC, and SCLC cells. (**D**) Representative images of immunostaining with anti-IL-17D antibody in tissue sections of human lung tumor adjacent tissue (**a**), NSCLC tissue (**b**) and SCLC tissue (**c**). Scale bars are 20 μm. (**E**) The frequency of cases with no (0), low (0.1–3.9), or high (4.0–9.0) IL-17D staining stratified by immunohistochemically-defined lung cancer subtype. (**F**) The frequency of cases with no (0), low (0.1–3.9), or high (4.0–9.0) IL-17D staining stratified by tumor stage. (**G**) Kaplan-Meier survival rates for 66 subjects with lung cancer disease with low (staining scores < 2, *n* = 46, blue line) versus high (staining scores ≥ 2, *n* = 20, red line) IL-17D expression were compared. Median survivals were undefined months (low IL-17D) versus 34.52 months (high IL-17D; *P* = 0.0309).

To identify genes and pathways participating in lung cancer development and progression, we analyzed the publicly available NCBI GEO dataset GSE32036 (expression profiling of lung cancer cell lines, including 59 normal small airway or bronchial epithelial cell samples, 118 NSCLC samples, and 29 SCLC samples) [13, 14]. A set of 710 genes (856 transcripts) showed significant expression differences between the normal, NSCLC, and SCLC groups (Figure 1A). Many well-known lung tumorigenesis-related genes were significantly enriched in SCLC, such as *GPC2*, *POU3F2*, *ASCL1*, *SYP* and *CXCR4*. Among these differently expressed genes, *IL-17D* gene, whose function in lung cancer is unknown, was upregulated in NSCLC and SCLC compared with that in normal airway epithelial cells (Figure 1B). We also analyzed single cell RNA-seq data of 42 NSCLC samples in NCBI GEO dataset GSE148071, and found that IL-17D was mainly expressed in lung cancer cells, while lowly expressed or not expressed in fibroblasts, alveolar cells, normal epithelial cells and immune cells (Supplementary Figure 1). Additional western blotting experiments were performed to verify the aberrant expression of IL-17D in these lung cancer cell lines; IL-17D was expressed at low or undetectable levels in HBECs and some NSCLC cell lines (H460, HCC827 and A549) but was highly expressed in all SCLC cell lines and H1155, an NSCLC cell line with neuroendocrine features (Figure 1C). ELISA analysis confirmed that the release of IL-17D in the supernatants was significantly elevated in SCLC cell lines and H1155 compared with A549 (Supplementary Figure 2). These data suggest that IL-17D expression is upregulated in lung cancer cells.

Subsequently, we performed immunohistochemical staining to study IL-17D expression in human lung cancer tissues, including 197 NSCLCs (50 squamous cell carcinomas, 124 adenocarcinomas, 12 adenosquamous carcinomas and 11 large cell carcinomas), 13 SCLCs and 68 normal lung tissues. IL-17D was detected at lower levels in normal lung tissues than in NSCLC and SCLC tissues (Figure 1D). Many IL-17D-positive lung cancer cells exhibited cytoplasmic localization (Figure 1D). Quantification of staining based on the intensity of IL-17D staining and the percentage of IL-17D-positive lung cancer cells revealed higher expression of IL-17D in SCLCs than in NSCLCs (Figure 1E). A higher IL-17D staining intensity was associated with the tumor stage of NSCLC (Figure 1F). To assess the prognostic significance of IL-17D, we analyzed the correlation between IL-17D expression and patient survival. Patients with a high density of IL-17D (staining scores ≥ 2, *n* = 20) had poorer overall survival than those with a low density of IL-17D (staining scores < 2, *n* = 46, *P* = 0.0309; Figure 1G). These results indicate that IL-17D is frequently overexpressed in lung cancers, and that a high expression level predicts poor prognosis in human lung cancer.

### IL-17D overexpression does not affect the biological behavior of tumor cells *in vitro*, but promotes tumor progression *in vivo*

**Figure 2:**
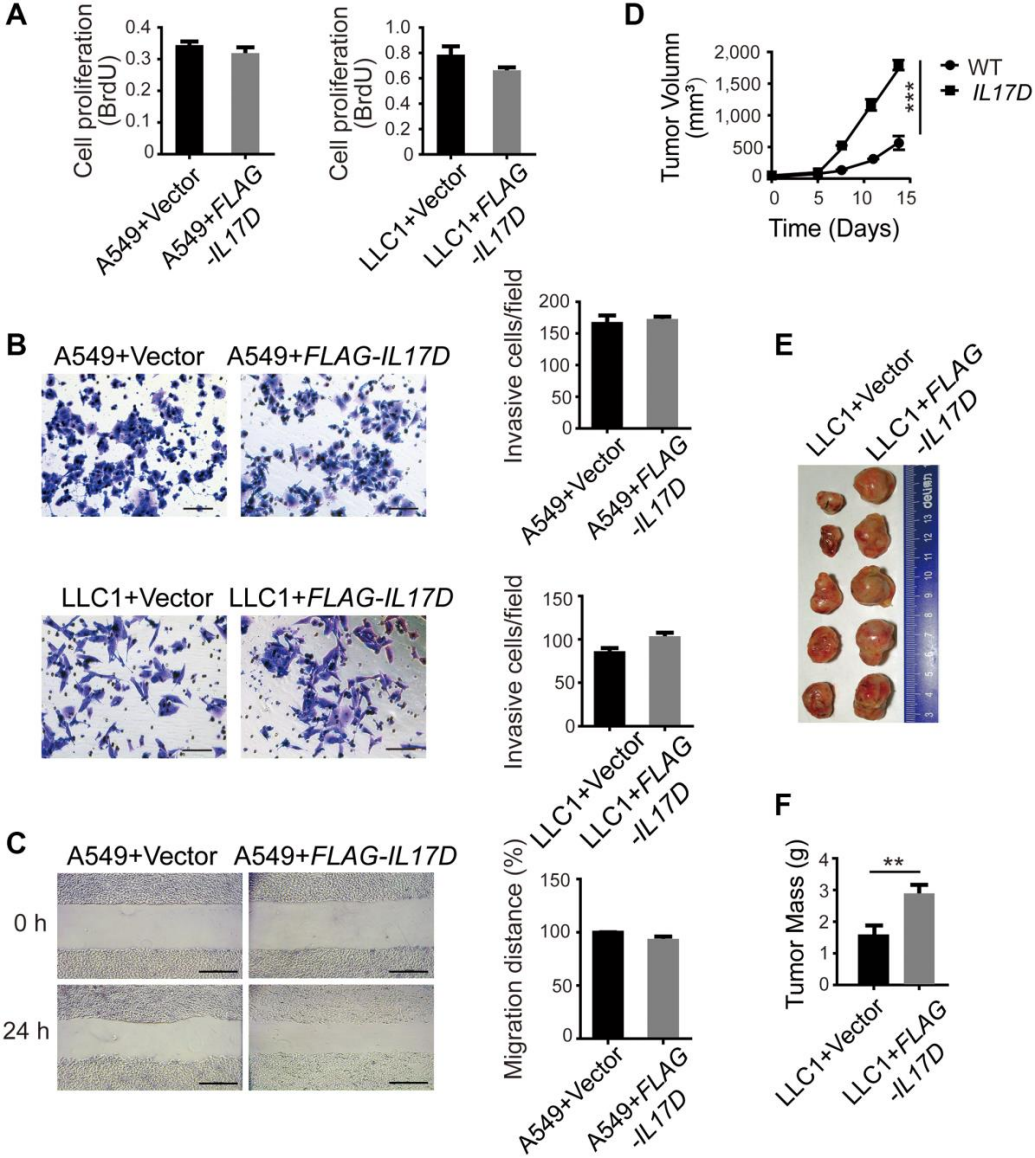
**IL-17D overexpression does not affect the biological behavior of tumor cells *in vitro*, but promotes tumor progression *in vivo*.** (**A**) Cell proliferation measurements (BrdU) compared between IL-17D–expressing and control cells (*n* = 5–6). (**B**) *IL-17D*–expressing cancer cells and control cells were subjected to invasion assay. Error bars represent the means ± standard deviation (SD) for a representative experiment performed in triplicate. Scale bars, 100 μm. (**C**) *IL-17D*–expressing A549 cells and control cells were subjected to a wound healing assay. Error bars represent the means ± SD for a representative experiment performed in triplicate. Scale bar, 100 μm. (**D**) Subcutaneous tumor growth of *IL-17D–*expressing and control LLC1 cells were measured (*n* = 5). Control and *IL-17D*–expressing LLC1 cell lines were harvested and injected into WT mice (100% tumor positive). Data are representative of three independent experiments. ^***^P < 0.001. (**E**) Images of subcutaneous tumors. Tumors were excised at day 15 after subcutaneous injection. (**F**) Subcutaneous tumor weight was measured and compared. ^**^P < 0.01.

To evaluate the function of IL-17D in lung cancer, we overexpressed IL-17D in the human lung carcinoma cell line A549 and mouse lung carcinoma cell line LLC1, which do not express endogenous IL-17D. We found that cell proliferation was slightly decreased in IL-17D–expressing cells (Figure 2A, no difference). We tested the effect of IL-17D expression on the invasion and migration of lung cancer cells. IL-17D expression also did not affect the invasion of A549 and LLC1 cells or cell migration (Figure 2B and 2C). These results suggest that IL-17D had no effect on the biological behavior of lung cancer cells *in vitro*.

To examine the effect of IL-17D expression on tumor growth *in vivo*, we subcutaneously injected LLC1 cells into 8-week-old C57BL/6 mice and measured the tumor size every 3 days. We found that subcutaneous tumor growth of IL-17D–expressing LLC1 cells was faster than that of vector-transduced cells (Figure 2D). On day 15, the tumors were excised, and the size of IL-17D–expressing tumors was larger than that of control tumors. (Figure 2E). The tumor weight of IL-17D–expressing cells was also significantly higher than that of the vector-transduced cells (Figure 2F). Owing to the different growth trends *in vitro* and *in vivo*, the major function of IL-17D in tumor growth might be associated with the tumor microenvironment (TME).

### IL-17D promotes infiltration of tumor-associated macrophages (TAMs)

**Figure 3:**
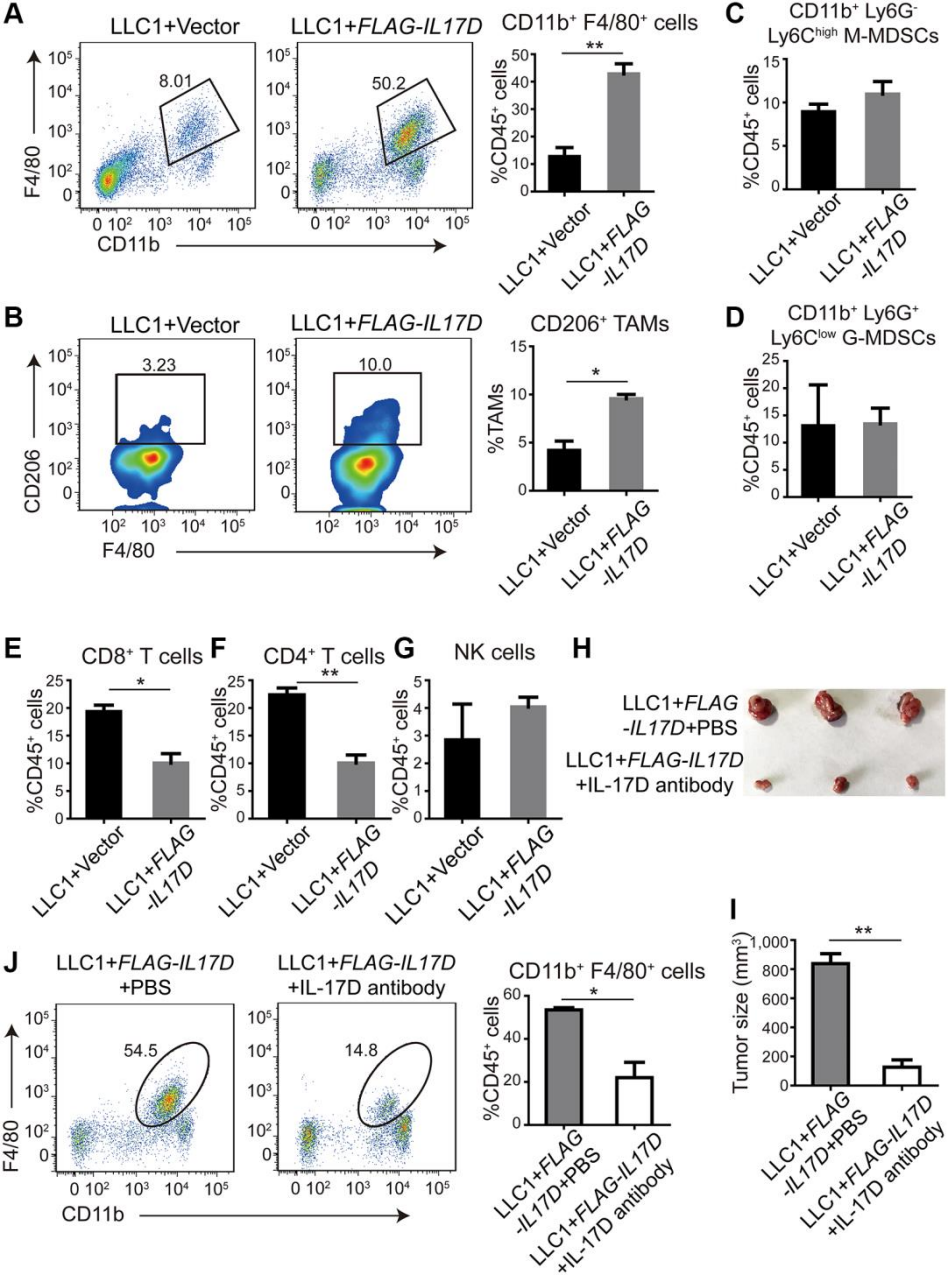
**IL-17D promotes infiltration of tumor-associated macrophages (TAMs).** (**A**) Representative flow cytometry profiles and percentages of TAMs in CD45+ TILs in subcutaneous tumors (*n* = 3). Mean ± SD. ^**^P < 0.01. (**B**) Representative flow cytometry profiles and percentages of CD206+ cells in CD11b+ F4/80+ cell populations in subcutaneous tumors (*n* = 2). Mean ± SD. ^*^P < 0.05. (**C** and **D**) Flow cytometric analysis and quantification of M-MDSCs (**C**) and G-MDSCs (**D**) in CD45+ TILs in subcutaneous tumors (*n* = 3). Mean ± SD. (**E**, **F**) Flow cytometric analysis and quantification of CD8+ T cells (**E**) and CD4+ T cells (**F**) in CD45+ TILs in subcutaneous tumors (*n* = 3). Mean ± SD. ^*^P < 0.05. ^**^P < 0.01. (**G**) Flow cytometric analysis and quantification of NK cells in CD45+ TILs in subcutaneous tumors (*n* = 3). Mean ± SD. (**H**) Images of subcutaneous tumors. (**I**) Subcutaneous tumor size was measured and compared. ^**^P < 0.01. (**J**) Representative flow cytometry profiles and percentages of TAMs in CD45+ TILs in subcutaneous tumors (*n* = 2). Mean ± SD. ^*^P < 0.05.

According to previous reports, IL-17D can induce endothelial cells to express IL-6, IL-8, and GM-CSF, which are involved in the differentiation and recruitment of myeloid cells in the TME [15–17]. To investigate the role of IL-17D in the immune microenvironment of lung cancer, we used flow cytometry to characterize the tumor-infiltrating immune cells in a subcutaneous tumor model of *IL17D*–expressing LLC1 cells. We found that the percentage of F4/80^+^ CD11b^+^ TAMs among CD45^+^ tumor-infiltrating leukocytes (TILs) was significantly increased in *IL17D*–expressing tumors (Figure 3A). M2 macrophages (CD206^+^ F4/80^+^ CD11b^+^ TAMs) in total TAMs were also markedly increased in *IL17D*–expressing tumors (Figure 3B). However, no significant changes in the percentage of myeloid-derived suppressor cells were observed in *IL17D*–expressing tumors compared to that in control tumors (Figure 3C and 3D). The percentages of CD8^+^ and CD4^+^ T cells in CD45^+^ TILs were significantly reduced in *IL17D*–expressing tumors (Figure 3E and 3F). The percentages of NK cells remained similar in *IL17D*–expressing and control tumors (Figure 3G and Supplementary Figure 3). To further study whether blocking IL-17D influences tumor progression via promoting TAM infiltration, we treated animals with an IL-17D antibody in subcutaneous mouse models. IL-17D–induced tumor growth and infiltration of TAMs were significantly inhibited by the IL-17D antibody (Figure 3H–3J). These results suggest that IL-17D promotes lung cancer progression by inducing TAM infiltration.

**Figure 4:**
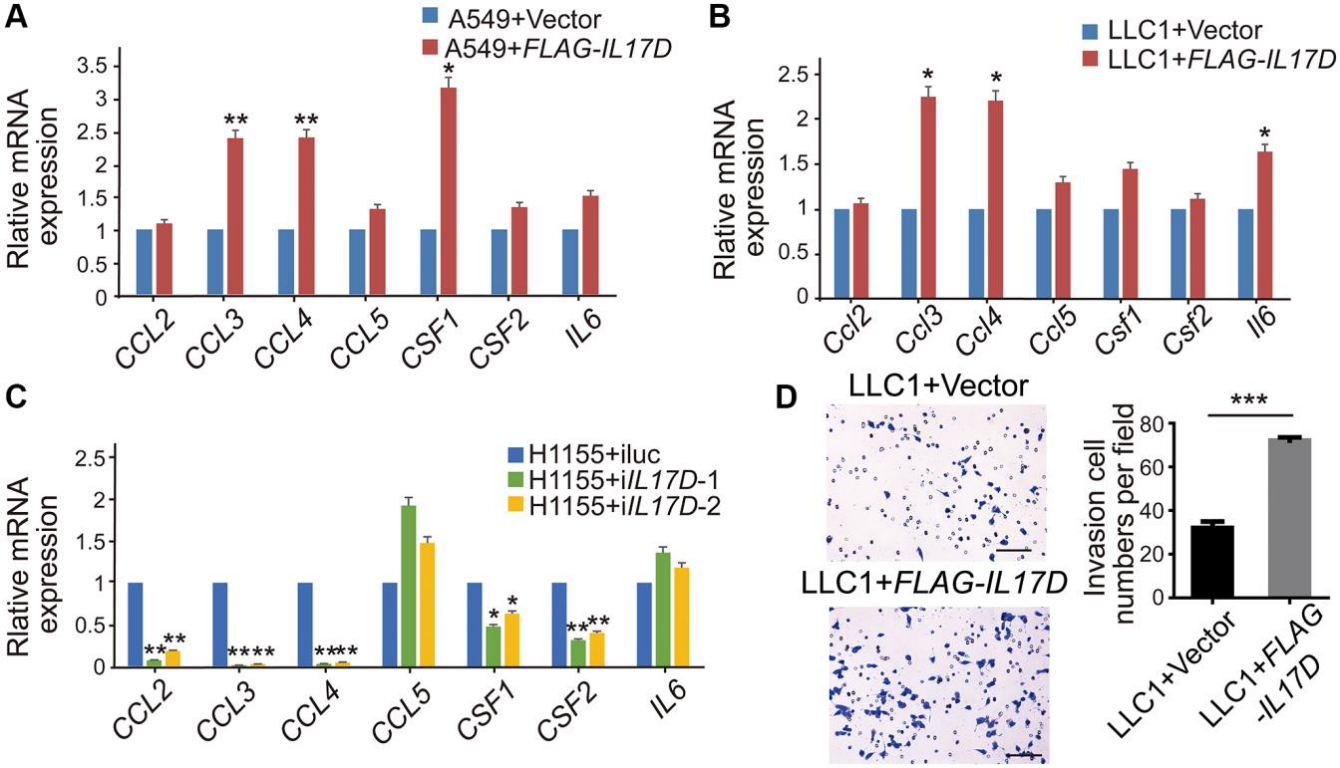
**IL-17D induces lung cancer cells to express a series of TAM recruitment– and polarization–related genes.** (**A**–**C**) The relative mRNA levels of genes related to macrophages recruitment and polarization was measured via quantitative real-time PCR. RNA was purified from A549 cells expressing IL-17D or empty vector (**A**), LLC1 cells expressing IL-17D or empty vector (**B**), and H1155 cells with knockdown of IL-17D or control (**C**). Mean ± SD. ^*^P < 0.05. ^**^P < 0.01. (**D**) Representative images and quantification of recruited macrophages. Mean ± SD. ^***^P < 0.001. Scale bars, 100 μm.

We next examined the expression of well-known genes related to macrophage recruitment and polarization, including *CCL2, CCL3, CCL4, CCL5, CSF1, CSF2* and *IL6* [15, 17, 18]. The mRNA expression of *CCL3, CCL4*, and *CSF1* was significantly upregulated in *IL17D*–expressing A549 cells compared with that in control cells (Figure 4A), whereas *IL17D* overexpression in the murine lung cancer cell line LLC1 increased *Ccl3, Ccl4*, and *Il6* expression (Figure 4B). ELISA assay showed that the release of CCL3 and CCL4 in the A549 cell supernatants was significantly elevated by IL-17D overexpression (Supplementary Figure 4). Consistently, *IL17D* knockdown in H1155 cells caused reciprocal changes in the expression of *CCL3, CCL4*, and *CSF1* compared with *IL17D* expression in A549 cells (Figure 4C), suggesting that IL-17D expression might generally induce macrophage recruitment– and polarization–related gene expression. Furtherly we performed a recruitment assay to confirm the effect of IL-17D on macrophage infiltration *in vitro*. As shown in Figure 4D, the recruitment of macrophages was significantly increased in *IL17D*–expressing LLC1 cells compared with that in control cells. These data indicate that IL-17D could induce tumor cells to express a series of TAM recruitment– and polarization–related genes and promote infiltration of TAMs in lung cancer.

### IL-17D activates p38 MAPK signaling pathway in lung cancer

**Figure 5:**
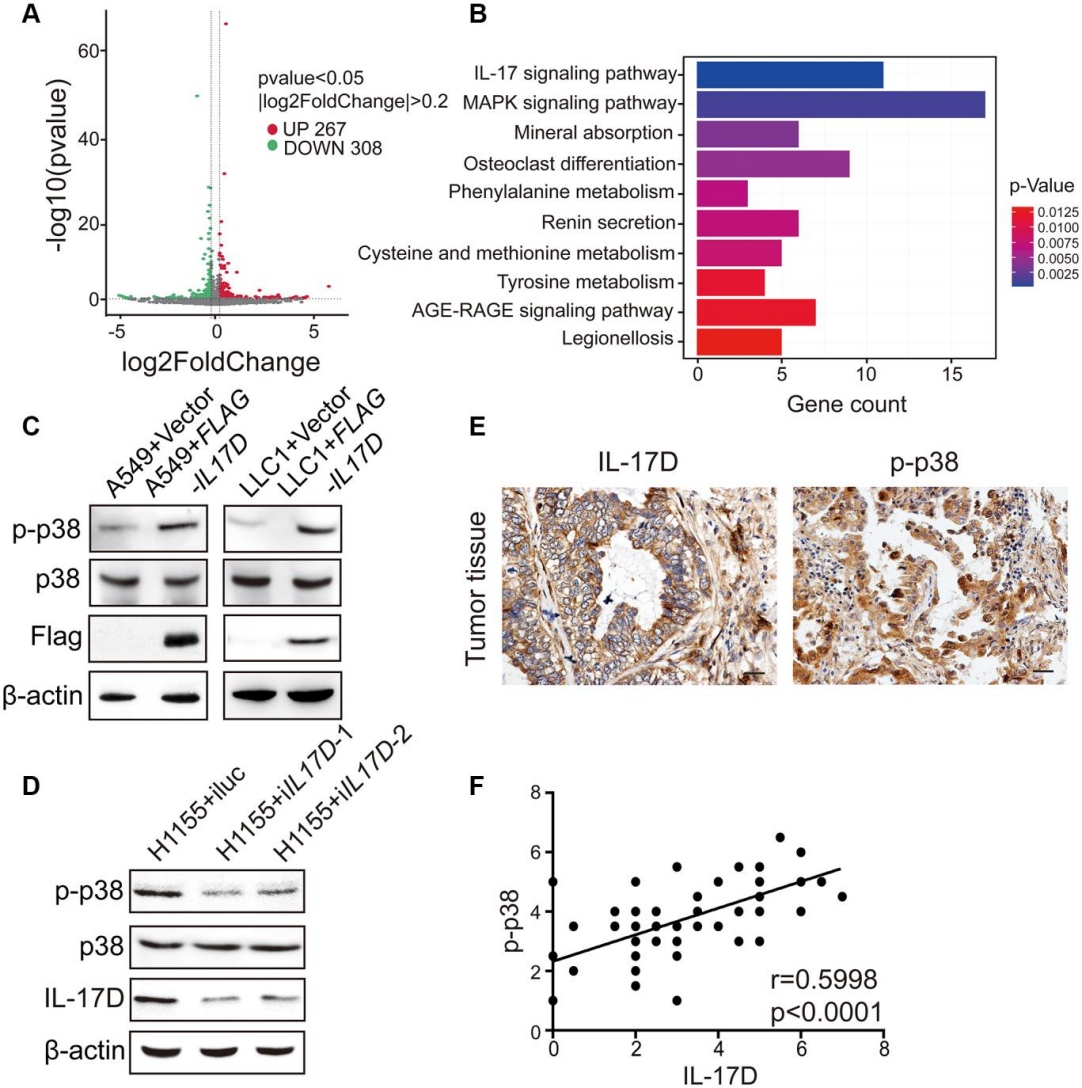
**IL-17D activates p38 MAPK signaling pathway in lung cancer.** (**A**) Volcano plot assessment of mRNA expression in A549 cells between IL-17D overexpression and control groups. (**B**) Significantly enriched KEGG pathways relative to differentially expressed mRNAs upon IL-17D overexpression. Y-axis represents pathways; X-axis represents the amount of the mRNAs enriched in KEGG pathways. (**C**) Immunoblots showing the expression of p38, p-p38, Flag and β-actin. Left, A549 cells were transduced with IL-17D or empty vector. Right, LLC1 cells were transduced with IL-17D or empty vector. (**D**) Immunoblots showing the expression of p38, p-p38, IL-17D and β-actin. H1155 cells were transduced with control or shRNAs against IL-17D. (**E**) Immunostaining of IL-17D and p-p38 in human lung cancer tissues. Scale bars are 20 μm. (**F**) Semiquantitative analysis of immunostaining revealed that IL-17D scores were associated with p-p38 scores (r = 0.5998, *P* < 0.0001).

To better understand the molecular mechanisms by which IL-17D promotes TAM infiltration, we performed RNA-seq analyses of *IL17D*–overexpressing and control A549 cells. By comparing gene expression within each category, we identified 308 downregulated genes and 267 upregulated genes related to IL-17D overexpression (Figure 5A). KEGG pathway analysis revealed that these significantly altered genes were mostly enriched in IL-17 and MAPK signaling pathways (Figure 5B). Lysates of *IL17D*–overexpressing A549 and LLC1 cells were then analyzed for MAPK activation via western blotting using activation-specific antibodies. p38 MAPK phosphorylation was induced in *IL17D*–overexpressing A549 and LLC1 cells compared with that in control cells (Figure 5C), but IL-17D overexpression did not affect ERK MAPK phosphorylation in A549 cells (Supplementary Figure 5). Similarly, *IL17D* knockdown in H1155 cells resulted in decreased phosphorylation of p38 MAPK (Figure 5D). These data indicate that IL-17D activates the p38 MAPK signaling pathway in lung cancer cell lines.

To evaluate the correlation between IL-17D and the activation of the p38 MAPK signaling pathway in individual primary lung cancer tissues, we used immunohistochemistry to study the expression levels of IL-17D and phosphorylated p38 (p-p38) in serial sections of lung cancer samples. As shown in Figure 5E, p-p38 was localized to both the cytoplasm and nucleus, albeit primarily in the nucleus. A highly significant positive correlation was observed between IL-17D and p-p38 expression (Figure 5F). These results indicate that IL-17D induces p38 MAPK phosphorylation and activity in lung cancer tissues.

### IL-17D promotes TAM infiltration via activation of the p38 MAPK signaling pathway in lung cancer

**Figure 6:**
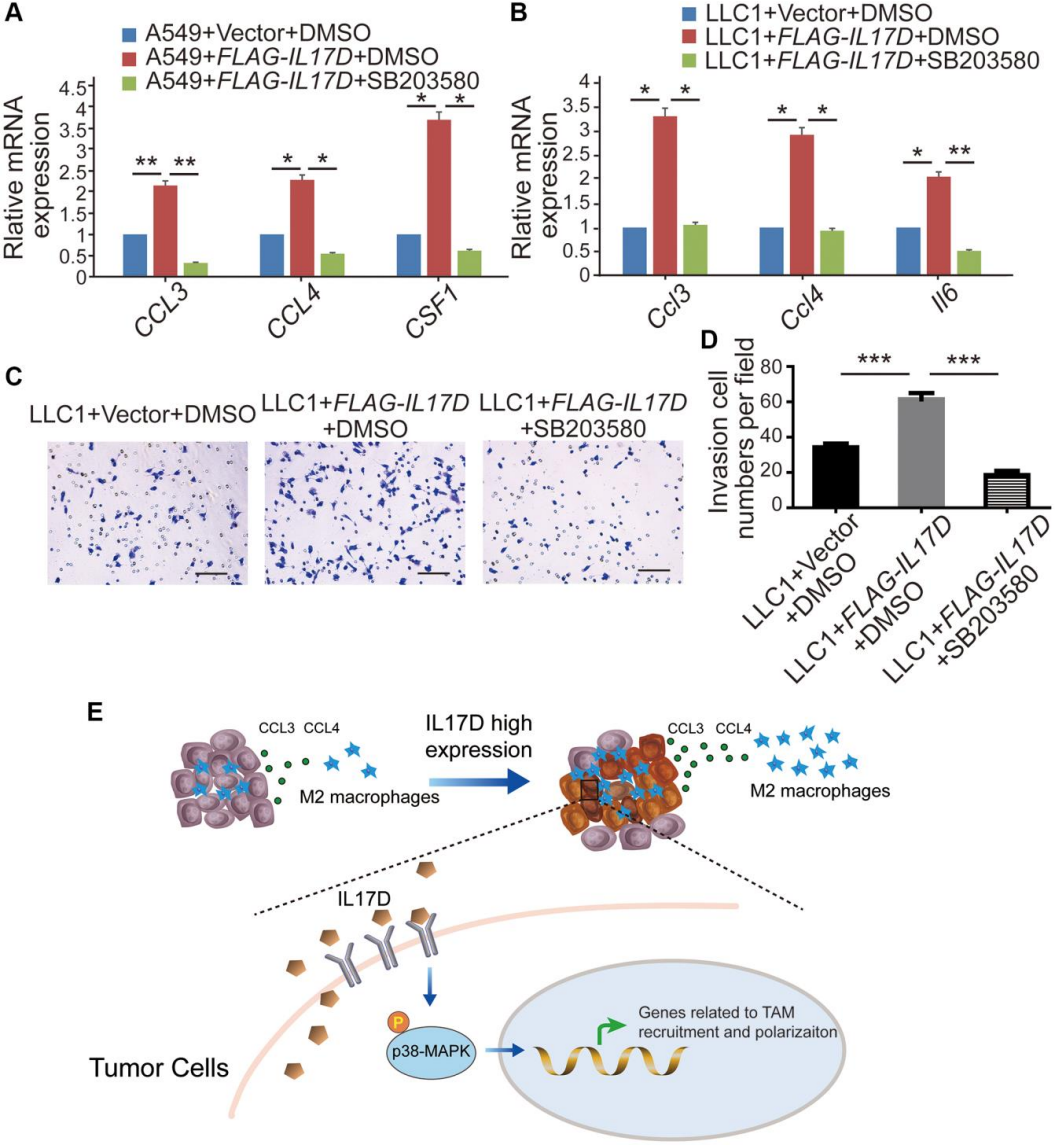
**IL-17D promotes TAM infiltration via the p38 MAPK signaling pathway in lung cancer.** (**A** and **B**) The relative mRNA levels of genes related to macrophages recruitment and polarization were measured via quantitative real-time polymerase chain reaction. RNA was purified from A549 cells expressing IL-17D treated with SB203580, IL-17D treated with DMSO, or empty vector treated with DMSO (**A**). RNA was purified from LLC1 cells expressing IL-17D treated with SB203580, IL-17D treated with DMSO, or empty vector treated with DMSO (**B**). Mean ± SD. ^*^P < 0.05. ^**^P < 0.01. (**C**) Representative images of recruited macrophages. Scale bars, 100 μm. (**D**) Quantification of recruited macrophages. Mean ± SD. ^***^P < 0.001. (**E**) Proposed model for mechanism of IL-17D inducing TAMs infiltration into the TME. High expression of IL-17D in lung cancer cells activates p38 MAPK signaling pathway through binding to receptor complexes of lung cancer cells, which upregulates TAM recruitment– and polarization–related genes expression and promotes infiltration of TAMs in lung cancer.

To assess whether IL-17D promotes TAM infiltration by inducing p38 MAPK activity in lung cancer, we first examined the effect of a p38 MAPK inhibitor on the expression of genes related to TAM recruitment and polarization. Treatment with the p38 inhibitor SB203580 (30 μM) significantly decreased the expression of CCL3, CCL4, and CSF1 in *IL17D*–overexpressing A549 cells (Figure 6A) and the expression of *Ccl3, Ccl4*, and *Il6* in *IL-17D*–overexpressing LLC1 cells (Figure 6B). Furthermore, SB203580 suppressed IL-17D induced TAM infiltration (Figure 6C and 6D). These data indicated that IL-17D promoted TAM infiltration by inducing p38 MAPK activity.

## DISCUSSION

The IL-17 cytokine family is involved in host defense against infection, inflammatory response, and cancer. Recent studies have shown that IL-17 promotes tumorigenesis in a wide range of organs, including the colon [19], liver [20], pancreas [21], lung [22], and skin [23, 24]. IL-17 promotes lung tumorigenesis via several different pathways, such as inducing MMP9 and MMP2 secretion to promote cancer cell invasion [25, 26], changing the expression of EMT-related markers [27], promoting CXCR-2-dependent angiogenesis [28], directly recruiting macrophages through IL-17 receptor A and IL-17 receptor C [29] and promoting M2 macrophage differentiation through COX-2/PGE2 pathway in the microenvironment of lung cancer [30]. IL-17D is a novel cytokine of the IL-17 family of cytokines whose function in lung cancer has not been elucidated. In this study, we found that aberrantly-expressed IL-17D was associated with lung cancer development and progression. IL-17D promoted primary tumor growth in a subcutaneous tumor model, whereas it did not affect lung cancer cell proliferation *in vitro*, indicating that IL-17D might promote tumorigenesis by influencing the tumor immune microenvironment.

To study the function of IL-17D in the TME, we used flow cytometry and discovered that IL-17D significantly increased the infiltration of TAMs into the TME. TAMs in lung cancer, as the most abundant immune cell component of TME, promote cancer proliferation, epithelial-mesenchymal transition, invasion, and metastasis [31], contributing to tumor development and exhibiting many characteristics of M2 macrophages. A previous study revealed that increased M2 macrophages are an important feature of SCLC [32], consistent with the high expression of IL-17D in SCLC. TAM-secreted IL-10 blunts CD8^+^ T cell responses and NK cell cytotoxicity by inhibiting DC production of IL-12 [33, 34]. In this study, IL-17D overexpression in a subcutaneous tumor model significantly inhibited CD8^+^ T cell infiltration. A previous report showed that IL-17D promotes the recruitment of NK cells through activating endothelial cells [11]. Our study indicated that IL-17D overexpression in subcutaneous tumor model of lung cancer exhibited a similar level of NK cells as control tumor. Furthermore, we found that a series of genes related to TAM recruitment and polarization, including *CCL3, CCL4, CSF1*, and *IL6*, were significantly upregulated by IL-17D. *CCL3* and *CCL4* are well-known chemotactic factors influencing tumor macrophage populations [18]. *CSF1* is expressed in multiple tumors and can recruit and induce immunosuppressive TAMs by binding and activating CSF1R to support tumorigenesis [35]. IL6, a pro-inflammatory cytokine produced by various cell types, promotes tumor progression by inducing the transformation of monocytes to the M2 phenotype [36]. We found that *CCL3, CCL4*, and *CSF1* were significantly upregulated in *IL17D*–expressing human lung cancer cells, whereas *Ccl3, Ccl4*, and *Il6* were induced in *IL17D*–expressing murine lung cancer cells. Huang et al. found that IL-17D could bind to CD93, a glycoprotein expressed on mature group 3 innate lymphoid cells (ILC3s), which regulates ILC3 maturation [37]. CD93 is expressed by various cells, including endothelial cells, myeloid cells, and even tumor cells in scRNA-seq dataset (GSE148071). These suggest that IL-17D might regulates lung cancer cells producing cytokines through CD93 on lung cancer cells, which indirectly leads to TAM infiltration. However, the detailed molecular mechanism of IL-17D-CD93 axis in myeloid, endothelial cells and tumor cells needs to be further demonstrated.

Analysis of the RNA-seq data revealed that IL-17D overexpression significantly influenced IL-17 and MAPK signaling pathways. Previous studies have shown that the IL-17 family members IL-17A and IL-17F can induce chemokine production in endothelial cells via the MAPK pathway [38]. There are three different MAPK signaling modules that mediate extracellular signals into the nucleus: ERK, JNK, and p38 kinase [39]. These MAPK signaling modules are known to play a central role in a variety of cellular activities, ranging from cell survival to cytokine expression. ERK activation can upregulate cyclin D1 expression, thereby enhancing the cell cycle [40, 41]. The JNK pathway is involved in both apoptosis and survival signaling [42], whereas p38 activation is linked to pro-inflammatory cytokine production [43]. In the current study, IL-17D overexpression in lung cancer cells did not affect ERK activation, which is consistent with the lack of change in cell proliferation between IL-17D–expressing lung cancer and control cells. We observed an increase in the phosphorylation of p38 in *IL17D*–expressing A549 cells and LLC1 cells, and a reciprocal change in the phosphorylation of p38 in H1155 cells with knockdown of *IL17D*. We also found the IL-17D induced the expression of *CCL3, CCL4, CSF1*, and *IL6*, and macrophage infiltration was significantly suppressed by SB203580. These data indicate that IL-17D promotes TAM infiltration in a manner dependent on p38 MAPK activity.

Taken together, we propose a model regarding the role of IL-17D inducing TAM infiltration into TME. High expression of IL-17D in lung cancer cells activates p38 MAPK signaling pathway through binding to receptor complexes of lung cancer cells. The expression of genes related to TAM recruitment and polarization is significantly upregulated in the lung cancer cells with high expression of IL-17D, which finally promotes TAM infiltration (Figure 6E). Our study provides a rationale for understanding the function of IL-17D in tumor progression and suggests that targeting IL-17D and blocking p38 MAPK activity may be a new strategy for the treatment of lung cancer.

## MATERIALS AND METHODS

### Cell culture

Human bronchial epithelial cells (HBECs) immortalized with hTERT and CDK4 were obtained from Dr. Jerry Shay (UT Southwestern Medical Center, Dallas, TX, USA) in 2008. A549, H460, HCC827, H1155, H69, H526, H82, and mouse-derived Lewis lung carcinoma (LLC1) cell lines were obtained from the American Type Culture Collection (Manassas, VA, USA). RAW264.7 cells were purchased from Shanghai Zhong Qiao Xin Zhou Biotechnology Co., Ltd. (Shanghai, China). HBECs were maintained in serum-free keratinocyte medium (Gibco, Carlsbad, CA, USA). A549, H460, HCC827, H69, H526, and H82 cells were maintained in Roswell Park Memorial Institute 1640 medium (Biological Industries, Beit Haemek, Israel) supplemented with 10% fetal bovine serum (FBS, Biological Industries, Beit Haemek, Israel). H1155, LLC1 and RAW264.7 cells were maintained in Dulbecco’s modified Eagle medium (DMEM, Biological Industries, Beit Haemek, Israel) supplemented with 10% FBS. The cells were maintained in an incubator at 37°C in a humidified atmosphere containing 5% CO_2_.

### RNA-seq data

*IL-17D* gene expression was evaluated using NCBI Gene Expression Omnibus (GEO) datasets from studies comparing a large set of lung cancer cell lines (GSE32036) [13, 14] and human clinical sample-derived non-small cell lung carcinoma (NSCLC) cell lines vs. transformed small cell lung carcinoma (SCLC) cell lines (GSE64322) [44]. We analyzed this expression data using hierarchical clustering. Expression variability was assessed in The Cancer Genome Atlas studies of various cancer types.

### Sing-cell RNA-seq (scRNA-seq) analysis

The published data used for our analysis was obtained from the NCBI GEO database accession code GSE148071 [45]. The processed scRNA-seq expression matrix was downloaded for our analysis. Cells that had either lower than 200 or higher than 5000 expressed genes were removed. And cells with more than 30,000 UMIs and mitochondria content higher than 30% were discarded. A total of 89,612 cells were obtained for the downstream analysis. Seurat suite version 3.1 was used to perform cell clustering. Resolution 0.8 were used with FindClusters function to generate 38 cell clusters. 11 major cell types were identified by the differentially expressed genes (DEGs) of each cluster. To assign the 11 major cell types to each cluster, we scored each cluster by the normalized expressions of the marker genes selected by the researchers: Epithelial cells (*CAPS*, *SNTN*), Myeloid cells (*CD14*), Fibroblasts (*COL1A1*), T cells (*CD2*), B cells (*CD79A*), Alveolar cells (*CLDN18*), Neutrophils (*CSF3R*), Endothelial cells (*PECAM1*), Mast cells (*TPSB2*), Follicular dendritic cells (*FDCSP*). Cancer cell clusters were negative for normal lung epithelial markers and positive for *EPCAM*. The final results were visualized after the dimension reduction of UMAP (Uniform Manifold Approximation and Projection). Differentially expressed genes were identified using a Wilcoxon Rank Sum test by function FindMarkers of Seurat.

### Western blotting

Cells were lysed in RIPA buffer containing a phosphatase inhibitor (Solarbio, Beijing, China). The protein sample lysate was separated using a 10% sodium dodecyl sulfate (SDS)-polyacrylamide gel and transferred to a polyvinylidene fluoride membrane (Pall, Port Washington, NY, USA). Membranes were then immunoblotted with the following primary antibodies: anti-β-actin (Sigma-Aldrich, St. Louis, MO, USA, MAB1501, 1:5000), anti-FLAG (Sigma-Aldrich, F3165, 1:5000), anti-IL-17D (Abcam, Cambridge, UK, ab77185, 1:1000), anti-p38 MAPK (Cell Signaling Technology, CST, Danvers MA, USA, 9212, 1:1000), anti-Phospho-p38 MAPK (CST, 4511, 1:1000), anti-ERK (CST, 4695, 1:1000), anti-Phospho-ERK (CST, 4370, 1:1000). Proteins were visualized using Immobilon Western Chemiluminescent HRP substrate (Millipore, Burlington, MA, USA).

### Enzyme linked immunosorbent assay (ELISA)

The IL-17D, CCL3 and CCL4 levels were measured by enzyme-linked immunosorbent assay. Supernatant was harvested after 48 hours and centrifuged at 500g for 5 minutes at 4°C to pellet cells and debris. IL-17D concentrations were detected using Jiangsu Jingmei ELISA kit (JM-5561H2) according to the manufacturer’s instructions. CCL3 and CCL4 were measured using indicated ELISA kits obtained from MultiSciences (Hangzhou, China) (Human CCL3/MIP-1α ELISA Kit, EK161; Human CCL4/MIP-1β ELISA Kit, EK162) according to the manufacturer’s instructions. Absorbance was measured with a microplate reader at 450 nm wavelength to calculate the concentration of IL-17D, CCL3 and CCL4 in the sample.

### Immunohistochemical analysis

Lung cancer tissues were obtained from the Tianjin Medical University Cancer Institute and Hospital in China. The samples were fixed in 4% paraformaldehyde at 4°C overnight and embedded in paraffin. Paraffin blocks were cut into 5 μm sections, immunostained with antibodies against IL-17D (MyBioSource, San Diego, CA, USA. MBS9412960, 1:100) and Phospho-p38 MAPK (Thr180/Tyr182) (CST, 4511, 1:800), and processed following the standard protocol for DAB staining [46, 47]. The use of all human lung cancer tissues and clinical data was approved by the institutional review board of Tianjin Medical University. Informed consent was obtained in accordance with the principles of the Declaration of Helsinki. The samples were identified prior to analysis.

### Lentiviral transfection

H82 cDNA was used to amplify the full-length IL-17D open reading frame (ORF). *IL17D* was ligated into the lentiviral shuttle pCCL.PPT.hPGK.IRES.GFP/pre. DNA Oligos encoding an shRNA specific for IL-17D were ligated into pCCL.PPT.hPGK.GFP.Wpre vector. The primer sequences for the IL-17D ORF and the shRNA sequences targeting *IL17D* are listed in Supplementary Table 1.

These plasmids were used to produce lentivirus in HEK293T cells at a density of 80% in 100 mm dishes. Polyethylenimine (Polysciences, Warrington, PA, USA) was used as a transfection reagent, at a dosage one-third that of the plasmid.

### Cell proliferation assay

Cells (1 × 10^4^) were seeded into 96-well plates. Following incubation for 24 h at 37°C, a Cell Proliferation ELISA, BrdU (colorimetric) kit (Roche, Dublin, Ireland), was used to detect proliferation according to the manufacturer’s protocol [48].

### Expression profiling

Total RNA was isolated from exIL-17D and empty vector A549 cells using TRIzol (Invitrogen, Carlsbad, CA, USA) and sequenced using an Illumina HiSeq 2500 from Beijing Novogene Corporation. Functional profiling of *IL-17D*-related genes was performed using clusterProfiler and The Database for Annotation, Visualization, and Integrated Discovery (DAVID) based on the biological pathways from the Kyoto Encyclopedia of Genes and Genomes (KEGG) databases [49, 50].

### Invasion assay

Cells (2 × 10^5^) were plated without serum on 8 μm pore size Transwell filters (Corning, Corning NY, USA), coated with 20% growth factor–reduced Matrigel. Complete medium was placed in the bottom chambers as a chemoattractant. The chambers were incubated for 24 h at 37°C and 5% CO_2_. The migrated cells on the undersides of the filter membrane were fixed in 4% paraformaldehyde for 15 min at room temperature and stained with 0.5% crystal violet for 10 min. After washing the chambers three times in PBS, the cells at the top of the Matrigel membrane were removed using a cotton swab. The migrated cells were then counted under a light microscope.

### Wound healing assay

Cells were seeded into 6-well plates, incubated to achieve 90% confluence, and then scratched with a sterile 200 μL pipette tip. After rinsing with phosphate-buffered saline, cells were cultured in medium for 24 h at 37°C. Cell migration was observed and measured under a light microscope.

### Subcutaneous mouse models

Female C57BL/6 mice (6–8 weeks old) were obtained from Nanjing Biomedicine Research Institution (Nanjing, China). Tumor cell lines were harvested and subcutaneously injected into the right inguinal region of wild-type mice at a density of 1 × 10^6^ cells/mouse. Tumor size was measured every 3–4 days with a caliper and calculated according to the following formula: size = (π × length × width^2^)/6. The tumors were surgically removed 15 days after injection, and tumor weight was measured immediately. The experiment was repeated three times.

C57BL/6 mice were injected subcutaneously in the right inguinal region with 100 μL of anti-IL-17D polyclonal antibody (10 μg/mL, diluted with PBS) and PBS (control) every 4 days for 2 weeks, starting 2 days before the subcutaneous injection of IL-17D–overexpressing LLC1 cells. The tumors were surgically removed 13 days after injection of LLC1 cells. Tumor size was measured using a caliper and calculated.

### Tumor dissociation and cell isolation

Tumors were excised on day 15 after transplantation, cut into small pieces, and incubated in dissociation solution with 5 μg/mL collagenase type I (Sigma-Aldrich, St. Louis, MO, USA), 5 μg/mL hyaluronidase (Sigma-Aldrich), and 5 μg/mL DNase (Sigma-Aldrich). The solution was pipetted every 10 min during the incubation, and the suspension was dispersed through a 70 μm cell strainer. To identify cells from tumors, single-cell suspensions were stained with antibodies (BioLegend, San Diego, CA, USA). Live/dead cell discrimination was performed using Fixable Viability Stain 520 (Becton Dickinson and Company). Macrophages (CD45^+^ CD11b^+^ F4/80^+^ CD206^+^), monocytic-myeloid-derived suppressor cells (M-MDSCs, CD45^+^ CD11b^+^ ly6G^-^ ly6C^high^), granulocytic-myeloid-derived suppressor cells (G-MDSCs, CD45^+^ CD11b^+^ ly6G^+^ ly6C^low^), and T cells (CD45^+^ CD3^+^ CD4^+^ CD8^+^) were sorted using a BD FACSCanto II flow cytometer (Becton Dickinson and Company, Franklin Lakes, NJ, USA).

### Quantitative real-time PCR

Total RNA was isolated using TRIzol reagent (Invitrogen) according to the manufacturer’s instructions. cDNA was synthesized from 2 μg of total RNA using a cDNA Reverse Transcription Kit (Thermo Fisher Scientific, Waltham MA, USA). The target mRNA levels of cDNA were assessed by quantitative real-time PCR with SYBR Green PCR master Mix (DBI) in an ABI Prism 7900 system. We calculated the relative mRNA levels normalized to human GAPDH levels in the same samples. The primers used in this study are listed in Supplementary Table 1.

### Recruitment assays

RAW264.7 cells (1 × 10^5^) in DMEM medium were seeded into the upper chamber of 24-well Transwell inserts (8 μm pore size; Corning). Concentrated LLC1 supernatant containing chemoattractant was added to the lower chambers. Cells were incubated for 5 h in 5% CO_2_ at 37°C. The cells that migrated and attached to the lower surface of the Transwell membrane were fixed in 4% paraformaldehyde and stained with 0.5% crystal violet. After washing the chambers three times in PBS, the cells at the top of the membrane were removed using a cotton swab. Cells in five random fields were counted using a phase-contrast microscope (200× magnification) to analyze the migration rate.

### p38 MAPK inhibition

Cells (1 × 10^6^) were seeded in 6-well plates and treated with SB203580 (30 μM, MCE) at 37°C in 5% CO_2_. After 24 h, the cells were harvested for immunoblotting and qRT-PCR assays. For recruitment assays, cells were seeded in 100 mm plates and treated with DMSO or SB203580 in serum-free medium. After 12 h, the supernatant was collected and concentrated by centrifugation at 4000 rpm for 20 min using a Microsep 3 K Omega centrifugal filter (Pall). The supernatant was used in the recruitment assay.

### Statistical analysis

Data analysis was performed using the SPSS software v19.0. Kaplan-Meier analysis was used to compared overall survival and differences in survival were determined by the log-rank test. One-way analysis of variance and Student’s *t*-test were used for comparisons of means between groups, and Spearman's rho was used for correlation analysis. *P* < 0.05 was considered to be significant.

### Data availability

The RNA-seq data generated in this study were deposited in the GEO database (https://www.ncbi.nlm.nih.gov/geo/) under accession number GSE191111.

## Supplementary Materials

Supplementary Figures

Supplementary Table 1

## References

[1] Schreiber RD, Old LJ, Smyth MJ. Cancer immunoediting: integrating immunity's roles in cancer suppression and promotion. Science. 2011; 331:1565–70. 10.1126/science.1203486 21436444

[2] O'Donnell JS, Teng MWL, Smyth MJ. Cancer immunoediting and resistance to T cell-based immunotherapy. Nat Rev Clin Oncol. 2019; 16:151–67. 10.1038/s41571-018-0142-8 30523282

[3] Zitvogel L, Tesniere A, Kroemer G. Cancer despite immunosurveillance: immunoselection and immunosubversion. Nat Rev Immunol. 2006; 6:715–27. 10.1038/nri1936 16977338

[4] McGeachy MJ, Cua DJ, Gaffen SL. The IL-17 Family of Cytokines in Health and Disease. Immunity. 2019; 50:892–906. 10.1016/j.immuni.2019.03.021 30995505 PMC6474359

[5] Miossec P, Kolls JK. Targeting IL-17 and TH17 cells in chronic inflammation. Nat Rev Drug Discov. 2012; 11:763–76. 10.1038/nrd3794 23023676

[6] Blauvelt A, Chiricozzi A. The Immunologic Role of IL-17 in Psoriasis and Psoriatic Arthritis Pathogenesis. Clin Rev Allergy Immunol. 2018; 55:379–90. 10.1007/s12016-018-8702-3 30109481 PMC6244934

[7] Ruiz de Morales JMG, Puig L, Daudén E, Cañete JD, Pablos JL, Martín AO, Juanatey CG, Adán A, Montalbán X, Borruel N, Ortí G, Holgado-Martín E, García-Vidal C, et al. Critical role of interleukin (IL)-17 in inflammatory and immune disorders: An updated review of the evidence focusing in controversies. Autoimmun Rev. 2020; 19:102429. 10.1016/j.autrev.2019.102429 31734402

[8] Liu X, Sun S, Liu D. IL-17D: A Less Studied Cytokine of IL-17 Family. Int Arch Allergy Immunol. 2020; 181:618–23. 10.1159/000508255 32516792

[9] Fagerberg L, Hallström BM, Oksvold P, Kampf C, Djureinovic D, Odeberg J, Habuka M, Tahmasebpoor S, Danielsson A, Edlund K, Asplund A, Sjöstedt E, Lundberg E, et al. Analysis of the human tissue-specific expression by genome-wide integration of transcriptomics and antibody-based proteomics. Mol Cell Proteomics. 2014; 13:397–406. 10.1074/mcp.M113.035600 24309898 PMC3916642

[10] Starnes T, Broxmeyer HE, Robertson MJ, Hromas R. Cutting edge: IL-17D, a novel member of the IL-17 family, stimulates cytokine production and inhibits hemopoiesis. J Immunol. 2002; 169:642–6. 10.4049/jimmunol.169.2.642 12097364

[11] O'Sullivan T, Saddawi-Konefka R, Gross E, Tran M, Mayfield SP, Ikeda H, Bui JD. Interleukin-17D mediates tumor rejection through recruitment of natural killer cells. Cell Rep. 2014; 7:989–98. 10.1016/j.celrep.2014.03.073 24794441 PMC4084720

[12] Lee Y, Clinton J, Yao C, Chang SH. Interleukin-17D Promotes Pathogenicity During Infection by Suppressing CD8 T Cell Activity. Front Immunol. 2019; 10:1172. 10.3389/fimmu.2019.01172 31244826 PMC6562898

[13] Byers LA, Diao L, Wang J, Saintigny P, Girard L, Peyton M, Shen L, Fan Y, Giri U, Tumula PK, Nilsson MB, Gudikote J, Tran H, et al. An epithelial-mesenchymal transition gene signature predicts resistance to EGFR and PI3K inhibitors and identifies Axl as a therapeutic target for overcoming EGFR inhibitor resistance. Clin Cancer Res. 2013; 19:279–90. 10.1158/1078-0432.CCR-12-1558 23091115 PMC3567921

[14] Schuster K, Venkateswaran N, Rabellino A, Girard L, Peña-Llopis S, Scaglioni PP. Nullifying the CDKN2AB locus promotes mutant K-ras lung tumorigenesis. Mol Cancer Res. 2014; 12:912–23. 10.1158/1541-7786.MCR-13-0620-T 24618618 PMC4058359

[15] Zhou K, Cheng T, Zhan J, Peng X, Zhang Y, Wen J, Chen X, Ying M. Targeting tumor-associated macrophages in the tumor microenvironment. Oncol Lett. 2020; 20:234. 10.3892/ol.2020.12097 32968456 PMC7500051

[16] Tobin RP, Jordan KR, Kapoor P, Spongberg E, Davis D, Vorwald VM, Couts KL, Gao D, Smith DE, Borgers JSW, Robinson S, Amato C, Gonzalez R, et al. IL-6 and IL-8 Are Linked With Myeloid-Derived Suppressor Cell Accumulation and Correlate With Poor Clinical Outcomes in Melanoma Patients. Front Oncol. 2019; 9:1223. 10.3389/fonc.2019.01223 31781510 PMC6857649

[17] Ruffolo LI, Jackson KM, Kuhlers PC, Dale BS, Figueroa Guilliani NM, Ullman NA, Burchard PR, Qin SS, Juviler PG, Keilson JM, Morrison AB, Georger M, Jewell R, et al. GM-CSF drives myelopoiesis, recruitment and polarisation of tumour-associated macrophages in cholangiocarcinoma and systemic blockade facilitates antitumour immunity. Gut. 2022; 71:1386–98. 10.1136/gutjnl-2021-324109 34413131 PMC8857285

[18] Ruytinx P, Proost P, Van Damme J, Struyf S. Chemokine-Induced Macrophage Polarization in Inflammatory Conditions. Front Immunol. 2018; 9:1930. 10.3389/fimmu.2018.01930 30245686 PMC6137099

[19] Wang K, Kim MK, Di Caro G, Wong J, Shalapour S, Wan J, Zhang W, Zhong Z, Sanchez-Lopez E, Wu LW, Taniguchi K, Feng Y, Fearon E, et al. Interleukin-17 receptor a signaling in transformed enterocytes promotes early colorectal tumorigenesis. Immunity. 2014; 41:1052–63. 10.1016/j.immuni.2014.11.009 25526314 PMC4272447

[20] Ma S, Cheng Q, Cai Y, Gong H, Wu Y, Yu X, Shi L, Wu D, Dong C, Liu H. IL-17A produced by γδ T cells promotes tumor growth in hepatocellular carcinoma. Cancer Res. 2014; 74:1969–82. 10.1158/0008-5472.CAN-13-2534 24525743

[21] McAllister F, Bailey JM, Alsina J, Nirschl CJ, Sharma R, Fan H, Rattigan Y, Roeser JC, Lankapalli RH, Zhang H, Jaffee EM, Drake CG, Housseau F, et al. Oncogenic Kras activates a hematopoietic-to-epithelial IL-17 signaling axis in preinvasive pancreatic neoplasia. Cancer Cell. 2014; 25:621–37. 10.1016/j.ccr.2014.03.014 24823639 PMC4072043

[22] Chang SH, Mirabolfathinejad SG, Katta H, Cumpian AM, Gong L, Caetano MS, Moghaddam SJ, Dong C. T helper 17 cells play a critical pathogenic role in lung cancer. Proc Natl Acad Sci U S A. 2014; 111:5664–9. 10.1073/pnas.1319051111 24706787 PMC3992670

[23] Chen X, Cai G, Liu C, Zhao J, Gu C, Wu L, Hamilton TA, Zhang CJ, Ko J, Zhu L, Qin J, Vidimos A, Koyfman S, et al. IL-17R-EGFR axis links wound healing to tumorigenesis in Lrig1^+^ stem cells. J Exp Med. 2019; 216:195–214. 10.1084/jem.20171849 30578323 PMC6314525

[24] Wu L, Chen X, Zhao J, Martin B, Zepp JA, Ko JS, Gu C, Cai G, Ouyang W, Sen G, Stark GR, Su B, Vines CM, et al. A novel IL-17 signaling pathway controlling keratinocyte proliferation and tumorigenesis via the TRAF4-ERK5 axis. J Exp Med. 2015; 212:1571–87. 10.1084/jem.20150204 26347473 PMC4577838

[25] Li Q, Han Y, Fei G, Guo Z, Ren T, Liu Z. IL-17 promoted metastasis of non-small-cell lung cancer cells. Immunol Lett. 2012; 148:144–50. 10.1016/j.imlet.2012.10.011 23089548

[26] Shiau MY, Fan LC, Yang SC, Tsao CH, Lee H, Cheng YW, Lai LC, Chang YH. Human papillomavirus up-regulates MMP-2 and MMP-9 expression and activity by inducing interleukin-8 in lung adenocarcinomas. PLoS One. 2013; 8:e54423. 10.1371/journal.pone.0054423 23349885 PMC3549962

[27] Gu K, Li MM, Shen J, Liu F, Cao JY, Jin S, Yu Y. Interleukin-17-induced EMT promotes lung cancer cell migration and invasion via NF-κB/ZEB1 signal pathway. Am J Cancer Res. 2015; 5:1169–79. 26045995 PMC4449444

[28] Numasaki M, Watanabe M, Suzuki T, Takahashi H, Nakamura A, McAllister F, Hishinuma T, Goto J, Lotze MT, Kolls JK, Sasaki H. IL-17 enhances the net angiogenic activity and in vivo growth of human non-small cell lung cancer in SCID mice through promoting CXCR-2-dependent angiogenesis. J Immunol. 2005; 175:6177–89. 10.4049/jimmunol.175.9.6177 16237115

[29] Liu L, Ge D, Ma L, Mei J, Liu S, Zhang Q, Ren F, Liao H, Pu Q, Wang T, You Z. Interleukin-17 and prostaglandin E2 are involved in formation of an M2 macrophage-dominant microenvironment in lung cancer. J Thorac Oncol. 2012; 7:1091–100. 10.1097/JTO.0b013e3182542752 22534817 PMC3378786

[30] Li Q, Liu L, Zhang Q, Liu S, Ge D, You Z. Interleukin-17 Indirectly Promotes M2 Macrophage Differentiation through Stimulation of COX-2/PGE2 Pathway in the Cancer Cells. Cancer Res Treat. 2014; 46:297–306. 10.4143/crt.2014.46.3.297 25038765 PMC4132449

[31] Larionova I, Tuguzbaeva G, Ponomaryova A, Stakheyeva M, Cherdyntseva N, Pavlov V, Choinzonov E, Kzhyshkowska J. Tumor-Associated Macrophages in Human Breast, Colorectal, Lung, Ovarian and Prostate Cancers. Front Oncol. 2020; 10:566511. 10.3389/fonc.2020.566511 33194645 PMC7642726

[32] Hu X, Gu Y, Zhao S, Hua S, Jiang Y. Increased IL-10+CD206+CD14+M2-like macrophages in alveolar lavage fluid of patients with small cell lung cancer. Cancer Immunol Immunother. 2020; 69:2547–60. 10.1007/s00262-020-02639-z 32583155 PMC11027454

[33] Ruffell B, Chang-Strachan D, Chan V, Rosenbusch A, Ho CM, Pryer N, Daniel D, Hwang ES, Rugo HS, Coussens LM. Macrophage IL-10 blocks CD8+ T cell-dependent responses to chemotherapy by suppressing IL-12 expression in intratumoral dendritic cells. Cancer Cell. 2014; 26:623–37. 10.1016/j.ccell.2014.09.006 25446896 PMC4254570

[34] Peranzoni E, Lemoine J, Vimeux L, Feuillet V, Barrin S, Kantari-Mimoun C, Bercovici N, Guérin M, Biton J, Ouakrim H, Régnier F, Lupo A, Alifano M, et al. Macrophages impede CD8 T cells from reaching tumor cells and limit the efficacy of anti-PD-1 treatment. Proc Natl Acad Sci U S A. 2018; 115:E4041–50. 10.1073/pnas.1720948115 29632196 PMC5924916

[35] Lin W, Xu D, Austin CD, Caplazi P, Senger K, Sun Y, Jeet S, Young J, Delarosa D, Suto E, Huang Z, Zhang J, Yan D, et al. Function of CSF1 and IL34 in Macrophage Homeostasis, Inflammation, and Cancer. Front Immunol. 2019; 10:2019. 10.3389/fimmu.2019.02019 31552020 PMC6736990

[36] Toyoshima Y, Kitamura H, Xiang H, Ohno Y, Homma S, Kawamura H, Takahashi N, Kamiyama T, Tanino M, Taketomi A. IL6 Modulates the Immune Status of the Tumor Microenvironment to Facilitate Metastatic Colonization of Colorectal Cancer Cells. Cancer Immunol Res. 2019; 7:1944–57. 10.1158/2326-6066.CIR-18-0766 31554639

[37] Huang J, Lee HY, Zhao X, Han J, Su Y, Sun Q, Shao J, Ge J, Zhao Y, Bai X, He Y, Wang X, Wang X, Dong C. Interleukin-17D regulates group 3 innate lymphoid cell function through its receptor CD93. Immunity. 2021; 54:673–86.e4. 10.1016/j.immuni.2021.03.018 33852831

[38] Iyoda M, Shibata T, Kawaguchi M, Hizawa N, Yamaoka T, Kokubu F, Akizawa T. IL-17A and IL-17F stimulate chemokines via MAPK pathways (ERK1/2 and p38 but not JNK) in mouse cultured mesangial cells: synergy with TNF-alpha and IL-1beta. Am J Physiol Renal Physiol. 2010; 298:F779–87. 10.1152/ajprenal.00198.2009 20042461

[39] Zhang W, Liu HT. MAPK signal pathways in the regulation of cell proliferation in mammalian cells. Cell Res. 2002; 12:9–18. 10.1038/sj.cr.7290105 11942415

[40] Pagès G, Lenormand P, L'Allemain G, Chambard JC, Meloche S, Pouysségur J. Mitogen-activated protein kinases p42mapk and p44mapk are required for fibroblast proliferation. Proc Natl Acad Sci U S A. 1993; 90:8319–23. 10.1073/pnas.90.18.8319 8397401 PMC47347

[41] Lavoie JN, L'Allemain G, Brunet A, Müller R, Pouysségur J. Cyclin D1 expression is regulated positively by the p42/p44MAPK and negatively by the p38/HOGMAPK pathway. J Biol Chem. 1996; 271:20608–16. 10.1074/jbc.271.34.20608 8702807

[42] Pedram A, Razandi M, Levin ER. Extracellular signal-regulated protein kinase/Jun kinase cross-talk underlies vascular endothelial cell growth factor-induced endothelial cell proliferation. J Biol Chem. 1998; 273:26722–8. 10.1074/jbc.273.41.26722 9756915

[43] Cuenda A, Rousseau S. p38 MAP-kinases pathway regulation, function and role in human diseases. Biochim Biophys Acta. 2007; 1773:1358–75. 10.1016/j.bbamcr.2007.03.010 17481747

[44] Niederst MJ, Sequist LV, Poirier JT, Mermel CH, Lockerman EL, Garcia AR, Katayama R, Costa C, Ross KN, Moran T, Howe E, Fulton LE, Mulvey HE, et al. RB loss in resistant EGFR mutant lung adenocarcinomas that transform to small-cell lung cancer. Nat Commun. 2015; 6:6377. 10.1038/ncomms7377 25758528 PMC4357281

[45] Wu F, Fan J, He Y, Xiong A, Yu J, Li Y, Zhang Y, Zhao W, Zhou F, Li W, Zhang J, Zhang X, Qiao M, et al. Single-cell profiling of tumor heterogeneity and the microenvironment in advanced non-small cell lung cancer. Nat Commun. 2021; 12:2540. 10.1038/s41467-021-22801-0 33953163 PMC8100173

[46] Du W, Xu X, Niu Q, Zhang X, Wei Y, Wang Z, Zhang W, Yan J, Ru Y, Fu Z, Li X, Jiang Y, Ma Z, et al. Spi-B-Mediated Silencing of Claudin-2 Promotes Early Dissemination of Lung Cancer Cells from Primary Tumors. Cancer Res. 2017; 77:4809–22. 10.1158/0008-5472.CAN-17-0020 28754672

[47] Li X, Xu Z, Du W, Zhang Z, Wei Y, Wang H, Zhu Z, Qin L, Wang L, Niu Q, Zhao X, Girard L, Gong Y, et al. Aiolos promotes anchorage independence by silencing p66Shc transcription in cancer cells. Cancer Cell. 2014; 25:575–89. 10.1016/j.ccr.2014.03.020 24823637 PMC4070880

[48] Heil J, Reifferscheid G. Detection of mammalian carcinogens with an immunological DNA synthesis-inhibition test. Carcinogenesis. 1992; 13:2389–94. 10.1093/carcin/13.12.2389 1473248

[49] Huang da W, Sherman BT, Lempicki RA. Systematic and integrative analysis of large gene lists using DAVID bioinformatics resources. Nat Protoc. 2009; 4:44–57. 10.1038/nprot.2008.211 19131956

[50] Kanehisa M, Furumichi M, Tanabe M, Sato Y, Morishima K. KEGG: new perspectives on genomes, pathways, diseases and drugs. Nucleic Acids Res. 2017; 45:D353–61. 10.1093/nar/gkw1092 27899662 PMC5210567

