# Supplementary material for: Interleukin-17D promotes lung cancer progression by inducing tumor-associated macrophage infiltration via the p38 MAPK signaling pathway
Source: Aging (Albany NY). 2022 Aug 5;14(15):6149–68. doi: 10.18632/aging.204208 (PMC9417222; doi:10.18632/aging.204208)
Supplement: Supplementary Figures [file aging-14-204208-s001.pdf]

SUPPLEMENTARY FIGURES

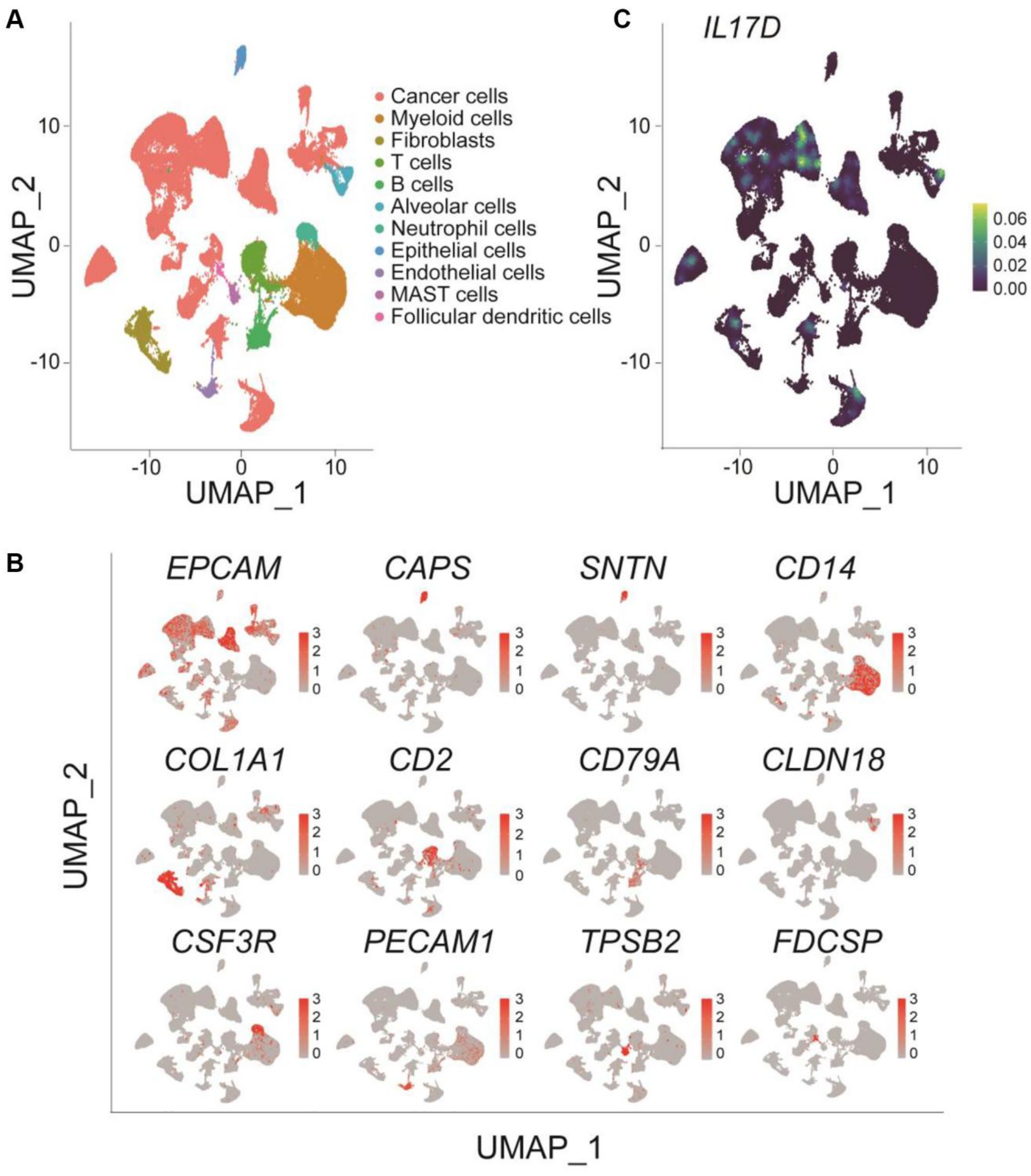

Supplementary Figure 1. Single cell RNA-seq analysis shows that IL-17D highly expresses in lung cancer cells, related to figure 1. (**A**) UMAP analysis shows transcriptionally differential clusters. Cells are colored by their 11 major cell types. (**B**) UMAP expression plots of known markers. (**C**) UMAP expression plots of *IL17D*. Scale bars represent z-score-normalized expression.

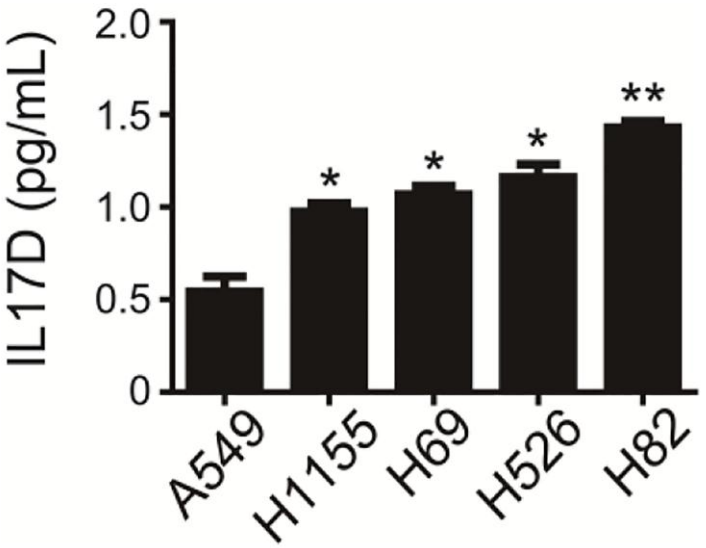

Supplementary Figure 2. IL-17D production in lung cancer cell lines was determined by ELISA, related to figure 1. Mean ± SD. $^{*}P < 0.05$. $^{**}P < 0.01$.

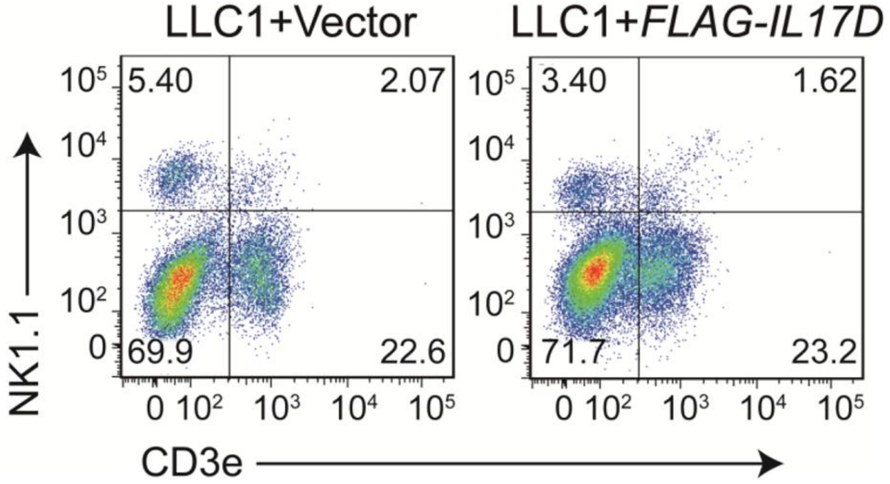

Supplementary Figure 3. Representative flow cytometry profiles of NK cells in CD45⁺ TILs in subcutaneous tumors, related to figure 3.

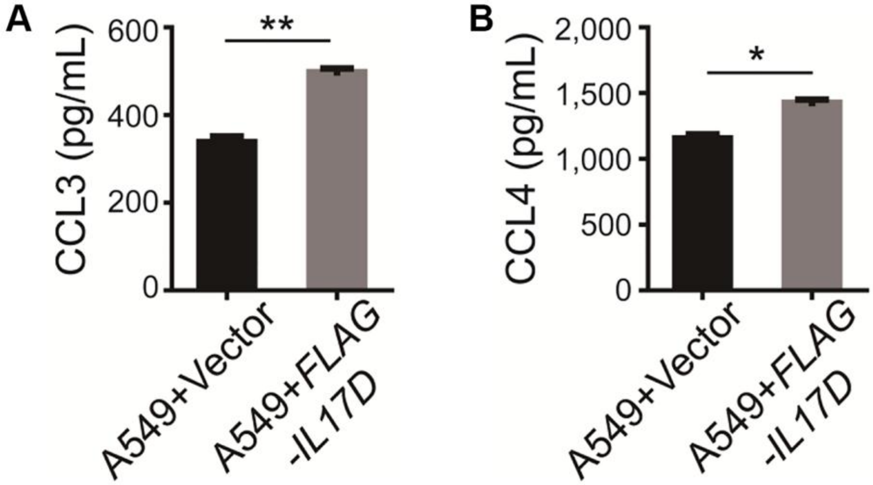

Supplementary Figure 4. IL-17D expression induces the release of CCL3 and CCL4 in A549 cell supernatants, related to figure 4. (**A**) CCL3 production was determined by ELISA in the supernatants of IL-17D–expressing and control A549 cells. (**B**) CCL4 production was determined by ELISA in the supernatants of IL-17D–expressing and control A549 cells. Mean ± SD. $^{*}P < 0.05$. $^{**}P < 0.01$.

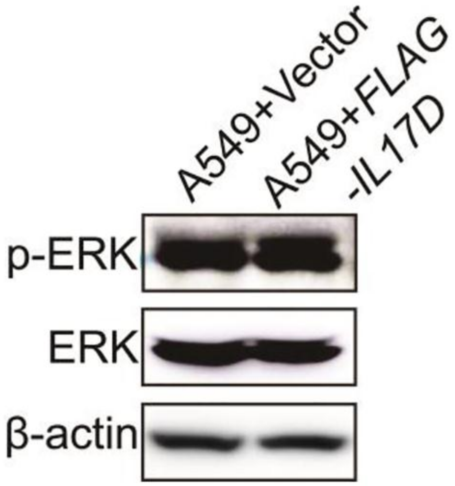

Supplementary Figure 5. IL-17D does not affect phosphorylation of ERK protein, related to figure 5. Immunoblots show expression of ERK, p-ERK and β-actin in A549 cells transduced with IL17D or empty vector.
