# Supplementary material for: Interleukin-17D promotes lung cancer progression by inducing tumor-associated macrophage infiltration via the p38 MAPK signaling pathway
Source: Aging (Albany NY). 2022 Aug 5;14(15):6149–68. doi: 10.18632/aging.204208 (PMC9417222; doi:10.18632/aging.204208)
Supplement: Supplementary Table 1 [file aging-14-204208-s002.pdf]

SUPPLEMENTARY TABLE

Supplementary Table 1. Primer sequences.

**Primers used for qRT-PCR**	
m*Gapdh*-F	5′ACTCCACTCACGGCAAATTCAACG3′
m*Gapdh*-R	5′TCTCGTGGTTCACACCCATCACAA3′
m*Csf1*-F	5′GCCTCCTGTTCTACAAGTGGAAG3′
m*Csf1*-R	5′ACTGGCAGTTCCACCTGTCTGT3′
m*Csf2*-F	5′CTCACCTGCTGCTACTCATTC3′
m*Csf2*-R	5′ACTACAGCTTCTTTGGGACAC3′
m*Ccl2*-F	5′GGAGAGCTACAAGAGGATCAC3′
m*Ccl2*-R	5′GTATGTCTGGACCCATTCCTTC3′
m*Ccl3*-F	5′GAAGATTCCACGCCAATTCATC3′
m*Ccl3*-R	5′GATCTGCCGGTTTCTCTTAGTC3′
m*Ccl4*-F	5′AGCTCTGTGCAAACCTAACC3′
m*Ccl4*-R	5′GGTGTAAGAGAAACAGCAGGAA3′
m*Ccl5*-F	5′GAGTATTTCTACACCAGCAGCA3′
m*Ccl5*-R	5′CCACTTCTTCTCTGGGTTGG3′
m*Il6*-F	5′TACCACTTCACAAGTCGGAGGC3′
m*Il6*-R	5′CTGCAAGTGCATCATCGTTGTTC3′
h*GAPDH*-F	5′GGTGGTCTCCTCTGACTTCAACA3′
h*GAPDH*-R	5′GTTGCTGTAGCCAAATTCGTTGT3′
h*CSF1*-F	5′TGAGACACCTCTCCAGTTGCTG3′
h*CSF1*-R	5′GCAATCAGGCTTGGTCACCACA3′
h*CSF2*-F	5′CTGCTGCTGAGATGAATGAAAC3′
h*CSF2*-R	5′CCCTTGAGCTTGGTGAGG3′
h*CCL2*-F	5′AGCAAGTGTCCCAAAGAAGC3′
h*CCL2*-R	5′CATGGAATCCTGAACCCACT3′
h*CCL3*-F	5′TGCATCACTTGCTGCTGACA3′
h*CCL3*-R	5′CTTCTGGACCCCTCAGGCACT3′
h*CCL4*-F	5′CTCATGCTAGTAGCTGCCTTC3′
h*CCL4*-R	5′GGC TGCTGGTCTCATAGTAATC3′
h*CCL5*-F	5′ACAGGTACCATGAAGGTCTCC3′
h*CCL5*-R	5′GCAAATTTGTGTAAGTTCAGG3′
h*IL6*-F	5′GGAGACTTGCCTGGTGAAA3′
h*IL6*-R	5′CTGGCTTGTTCCTCACTACTC3′
**Primers used for amplifying *IL17D* ORF**	
IL17D-ORF-F	5′CGGGATCCATGGATTACAAGGATGACGACGATAAGCTGGTAGCC GGCTTC3′
IL17D-ORF-R	5′CGGGATCCTCAGGGGCCAGCGGGCGCGT3′
**shRNA sequences for *IL17D***	
shIL17D-501	5′GATCCCCAGACAGCATCAACTCCAGCATTTCAAGAGAATGCTGG AGTTGATGCTGTCTTTTTTA3′
shIL17D-301	5′AGCTTAAAAAAGACAGCATCAACTCCAGCATTCTCTTGAAATGCT GGAGTTGATGCTGTCTGG G3′
shIL17D-502	5′GATCCCCCCCTGTCTACATGCCCACCGTTTCAAGAGAACGGTGGG CATGTAGACAGGGTTTTTA3′
shIL17D-302	5′AGCTTAAAAACCCTGTCTACATGCCCACCGTTCTCTTGAAACGGT GGGCATGTAGACAGGGGGG3′
